# Annual prevalence estimation of lymphatic malformation with a cutaneous component: observational study of a national representative sample of physicians

**DOI:** 10.1186/s13023-022-02336-3

**Published:** 2022-05-12

**Authors:** Jack Ray Gallagher, J. Martini, S. Carroll, A. Small, J. Teng

**Affiliations:** 1 Clarity Pharma Research LLC, 2375 E. Main Street, Suite A300, Spartanburg, SC 29307 USA; 2 Palvella Therapeutics Inc., 125 Strafford Avenue, Suite 360, Wayne, PA 19087 USA; 3 Department of Dermatology, Oregon Health and Science University, 3303 S. Bond Ave., Portland, OR 97239 USA; 4 Stanford University School of Medicine, Lucile Packard Children’s Hospital at Stanford, 700 Welch Road, Suite 301; MC5896, Stanford, CA 94304 USA

**Keywords:** Lymphatic malformations, Microcystic lymphatic malformations, Mixed lymphatic malformations, Cutaneous lymphatic malformations, Observational study, Prevalence, Epidemiology

## Abstract

**Background:**

Lymphatic malformations (LMs) represent a potentially life-threatening, rare disease of the lymphatic system characterized by development of abnormal vessels, outpouchings, or cysts filled with lymphatic fluid. There are three morphologic types of LMs based on the size of the individual cysts: macrocystic (typically > 2 cm), microcystic (generally < 2 cm), and mixed (includes aspects of both). Macrocystic LMs typically exist beneath the skin and often can involve vascular components and/or organs. Microcystic LMs often have a cutaneous component and clinically present with lymphorrhea, bleeding, pain, itching, malodor, and functional deficits. There are no treatments approved by the US Food and Drug Administration (FDA) for either macrocystic or microcystic lymphatic malformations. The totality of the epidemiologic literature for LM is limited to the incidence of the disease among various birth cohorts. This is the first nationally representative study to estimate the national managed prevalence for patients with microcystic LM or combined LM with a cutaneous component annually across physician specialties likely to manage this condition. We conducted a retrospective observational survey of a nationally representative sample of patient-care physicians in the United States most likely to manage lymphatic malformations with a cutaneous component (LMC). Once recruited, target physicians participated via an electronic questionnaire. We weighted study physician self-estimates of the number of LMC patients treated in the past 12 months to reflect the specialists’ corresponding proportion in the national universe. All patient information was anonymous; no personally identifiable information was collected.

**Results:**

Of the 420 physicians who visited the study website, 316 agreed to be screened and to participate (75.2% participation rate). Our survey results indicated the estimated number of unique annually managed LMC patients by target specialists is 79,920 (CI 66,600–93,250). This number corresponds to managed prevalence of 24.1 LMC patients per 100,000 population (CI 19.6/100,000–28.4/100,000).

**Conclusions:**

The study indicates that while rare, LMC affects a substantial number of people in the US (79,920) who are being managed by one or more specialists. By better understanding the prevalence of people living with LMC who require treatment, efforts to both increase disease awareness and to identify underserved populations in need of potential new treatments can be better focused.

## Background

Lymphatic malformations (LM) represent a serious, rare disease of the lymphatic system characterized by development of abnormal vessels, outpouchings, or cysts filled with lymphatic fluid. The distinguishing feature of this disease is the aberrant cellular growth that creates cysts of varying size and location. According to the International Society for the Study of Vascular Anomalies (ISSVA), there are three morphologic types of LMs based on the size of the individual cysts (as opposed to the overall size of the LM): macrocystic (typically > 2 cm), microcystic (generally < 2 cm), and mixed (includes aspects of both) [1]. Macrocystic LMs typically exist beneath the skin and often can involve vascular components and/or organs. Microcystic LMs often have a cutaneous component and clinically present with lymphorrhea, bleeding, pain, itching, malodor, and functional deficits. Infections of malformations can occur and may lead to cellulitis of surrounding tissues or severe, life-threatening infections.

There are no treatments approved by the US Food and Drug Administration (FDA) for either macrocystic or microcystic lymphatic malformations. The current standard of care treatment options are mainly limited to procedural interventions such as surgery and sclerotherapy [2]. Treatment success rates for surgery vary, especially for large infiltrative disease [3]. Sclerotherapy, which involves injection of a sclerosant into the vascular structure, is associated with a higher response rate of up to 75% in macrocystic LM but much less successful in microcystic LM [4]. Its use is limited by safety concerns including pulmonary fibrosis [5]. However, a small study by Chaudry and colleagues (n = 34) found that treatment with the sclerosant bleomycin for microcystic LM was relatively safe and effective [5]. Therefore, microcystic LM represents a high unmet need and serious genetic disease for which novel therapies should advance with a sense of urgency.

Within the past 10 years, researchers have identified that somatic activating mutations in phosphatidylinositol 3-kinase (PIK3CA) result in increased activation of the PIK3CA/AKT/mTOR signaling pathway, leading to increased lymphangiogenesis [6]. This discovery has allowed researchers to consider potential new therapies that target this cellular pathway. One therapy in particular that has shown promise in a clinical setting is the mTOR inhibitor sirolimus, which is not FDA-approved for lymphatic malformations [7, 8].

Because LM is a congenital disease, diagnosis and treatment often occur early in life at specialized vascular malformation or vascular anomaly treatment centers. Given the complexity of the disease, patients are cared for by a multi-disciplinary team of specialists at these centers, which may include providers from dermatology, hematology/oncology, plastic or general surgery, otolaryngology, radiology and interventional radiology, orthopedics, and genetics, among others.

The research to fully understand the prevalence of LM is sparse and does not differentiate between macrocystic and microcystic LM. The totality of the epidemiologic literature for LM (inclusive of macro and microcystic) is limited to the incidence of the disease among various birth cohorts. Although both macrocystic and microcystic LM are chronic diseases, macrocystic lesions have better responses to treatments with surgery or sclerotherapy. As microcystic lesions do not respond as well to these treatments, they tend to be more persistent; therefore indicating the need to study the prevalence of each LM subtype.

Previous studies report a birth incidence ranging from 1/250 live births to as few as 1/4000 live births [9, 10]. Applying these rates to the provisional 2020 US Centers for Disease Control and Prevention birth data would yield about 900–14,400 new incident cases in 2020 [11]. These studies and data are further challenged by the fact that the disease classification and nomenclature have gone through a number of iterations over the years and disease definitions across studies may not be aligned [12]. Given the different treatment approaches for macrocystic and microcystic LM, including the different success rates of therapy and outcomes, it is important to distinguish the prevalence of microcystic LM from macrocystic LM. The current data estimates of LM incidence fail to capture the prevalence of microcystic LM, as people with this disease continue to age and require chronic care and treatment. By better understanding the total prevalence of people living with microcystic LM in the United States, efforts to identify underserved populations in need of potential new treatments can be better focused.

The purpose of the present study was to obtain an accurate estimate of the national prevalence for patients with microcystic LM or combined LM managed annually across physician specialties likely to treat this condition. In our survey, we defined lymphatic malformations with a cutaneous component (LMC) as patients with either microcystic LM or mixed type LM.

This is the first study using a methodology designed to estimate the national prevalence of annually managed LMC. It was not designed to estimate the total national prevalence of LMC, but rather the prevalence of a particular subset—patients whose LMC is being managed by a physician in a target specialty.

## Results

Of the 420 randomly selected physicians who visited the study website, 316 agreed to be screened and to participate in the study (75.2% participation rate). Study physicians with LMC patients estimated 58% of their LMC patients treated in the past 12 months were co-managed by one or more additional physicians. Eighty-eight percent (88%) of study physicians were pediatricians, dermatologists, or hematologists/oncologists by primary specialty. The remaining physicians were pediatricians with subspecialties in either hematology/oncology or dermatology. The geographic distribution of study physicians by U.S. census region was statistically similar to the corresponding distribution of the universe of patient-care pediatricians, dermatologists, and hematology/oncologists in the U.S. (Northeast—22.5% vs. 23.9%, Midwest—20.1% vs. 21.5%, South—37.0% vs. 34.4%, and West—19.0% vs. 21.6%).

**Fig. 1:**
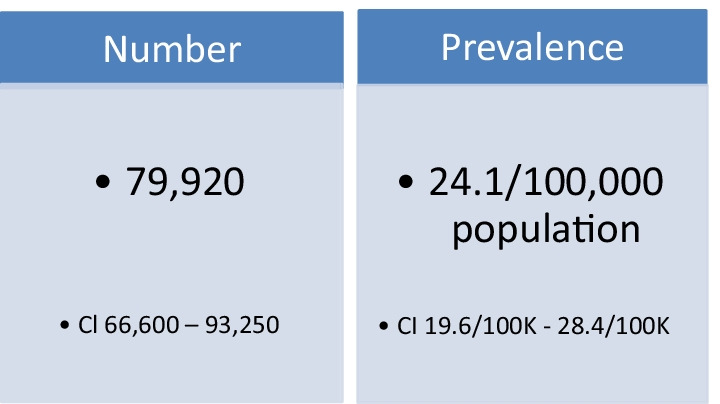
Estimated unique patients treated annually for LMC

Our survey results indicated that the estimated number of unique annually managed LMC patients by target specialists is 79,920 (CI 66,600–93,250). This number corresponds to managed prevalence of 24.1 LMC patients per 100,000 population (CI 19.6/100,000–28.4/100,000) (Fig. 1).

A majority of study physicians (218 of the total 316–69%) reported managing at least one diagnosed LMC patient during the previous 12 months, and 58% of these patients were co-managed with one or more physicians of a target specialty.

**Fig. 2:**
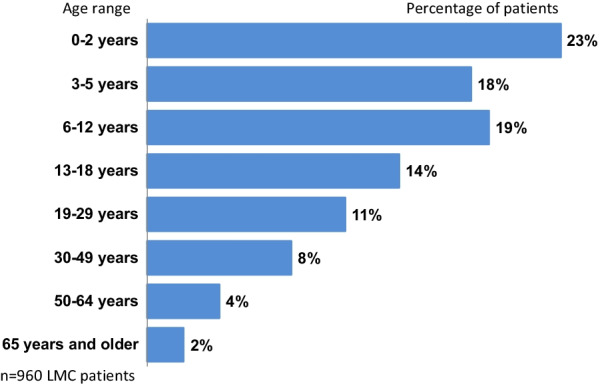
Estimated proportion of LMC by age group. The 218 physician survey participants who said they have LMC patients reported treating a total of 960 LMC patients currently, in the above age groups

Although this study shows that LMC is most frequently managed in pediatric patients, a fourth of study patients (25%) were 19 or older, and 6% were age 50 or older, showing that LMC may require treatment throughout one’s lifetime (Fig. 2).

## Discussion

A significant percentage of macrocystic and microcystic LMs are caused by somatic mutations in *PIK3CA* that result in abnormal lymph vessel formation. They differ, however, in disease presentation, clinical symptoms, treatment paradigms, and outcomes.

Our study is the first to use a nationally representative sample to estimate the treatment prevalence of LMC in the US. The prevalent population that we examined is the cohort of LM patients with a cutaneous component managed annually by the specialists/subspecialists often treating this condition (pediatricians, pediatric hematologists/oncologists, pediatric dermatologists, hematologists/oncologists, and dermatologists) (Table 1).

**Table 1:** Physician characteristics

	n = 316 total physicians	% of total (%)
*Specialty/subspecialty—n (%)*		
Pediatrics	102	32.3
Pediatric dermatology	9	2.8
Pediatric hematology/oncology	29	9.2
Dermatology	100	31.6
Hematology/oncology	76	24.1
*Practice setting—n (%)*		
Physician-owned private practice or private clinic	199	63.0
Academic/research hospital or associated outpatient clinic	65	20.6
Non-academic (community) hospital or associated outpatient clinic	41	13.0
Government/VA hospital/other	8	2.5
Specialized non-hospital-owned vascular clinic/center	3	0.9
*Number of unique total patients, all conditions, treated during past 12 months—n (%)*		
≤ 450	31	9.8
451–1199	117	37.1
1200–1799	100	34.5
≥ 1800	59	18.6

Mean: 1189; Median: 1200; Standard deviation: 527.2

Our estimated number of unique patients receiving LMC treatment annually is higher than past epidemiologic studies of LM because they focused solely on incidence at birth. Our study’s analytical model (Table 2) estimates that the annual treatment prevalence of LMC is 24.1 per 100,000 population (CI 19.6–28.4), representing 79,920 patients (CI 66,600–93,250) (Fig. 1).

**Table 2:** Estimates of annual LMC*-treated patients nationally

Specialty	MDs in study	MDs In US	LMCs/MD	LMCs in US	Unique total patients seen annually per MD	Unique total patients seen annually from national household study^d^	Adjustment for heavier than average patient load for specialty	Adjusted total
Pediatrics^a^	102	54,139	0.478	25,878	1217	NA	0	25,878
Pediatric dermatology^b,c^	9	625	5.000	3125	1456	NA	0	3125
Pediatric Hem/Onc^a^	29	2251	2.649	5963	729	NA	0	5963
Dermatology^a^	100	11,747	1.589	18,666	1556	1247	0.8014	14,959
Hematology/oncology^a^	76	11,255	2.665	29,995	811	NA	0	29,995
Total	316	80,017		83,627				79,920**

*Lymphatic malformation with cutaneous component − 93,250

**95% confidence interval 66,600

^a^Source: American Medical Association. AMA Physician Masterfile database, https://www.ama-assn.org. Accessed 5 May 2021

^b^Silverberg NB, MD. Pediatric dermatology workforce shortage explained. Cutis. 2018;102:305–06, cites about 300 board certified pediatric dermatologists, cites about double that total pediatric dermatologist practitioners, specifying “1 pediatric dermatologist for every 120,000 children or more”

^c^Prindaville B, Horii KA, Siegfried EC, Brandling-Bennett H. Pediatric dermatology workforce in the United States. Pediatr Dermatol. 2019;36:166–68, cites 283 board certified pediatric dermatologists

^d^Share of Americans who visited a dermatologist in the last 12 months in 2018, by age, Statista, https://www.statista.com/statistics/228530/people-who-used-a-skin-doctor-dermatologist-usa. Accessed 15 February 2022

**Fig. 3:**
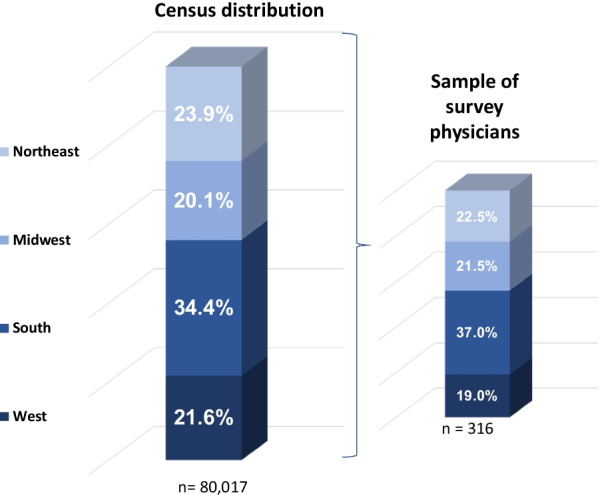
Universe of patient-care pediatricians, dermatologists, and Hem/Oncs by US Census Region. The smaller bar chart at right shows that the regional distribution of specialists participating in the survey closely aligns with the national distribution of these specialists, as listed in the American Medical Association’s “*Physician Characteristics and Distribution in the US, American Medical Association” (2015)*

Several study limitations should be considered when scrutinizing the above estimates. Among the most important is the extent to which study physicians are representative of all US physicians who treated LMC patients during the past 12 months. From a geographic perspective, the distribution of study physicians was statistically similar to the distribution of patient-care physicians in the target specialties in each US census region (Fig. 3). From an annual total patient load, which can affect the probability of treating an LMC patient, the mean annual number of unique patients seen was similar to the expected number for all but general dermatologists, who had a heavier than expected patient load for which we corrected by weighting down the number of LMC patients treated correspondingly.

Another potential limitation that could have led to significant underestimation of the number of treated adult patients is the extent to which LMC patients are treated by primary care or other specialty/subspecialty physicians who were not included in the study. This is a possibility because a survey of adult patients with vascular malformations (of which LM is a type) conducted by Blei et al. found that after “aging out” of the pediatric age group, these patients have great difficulties finding adequately educated adult-care physicians to take over their management, possibly because vascular anomalies remain an orphan field that is not covered in most medical schools or residency programs [12].

Other potential biases include sampling error, the corresponding ranges of which we noted above, and recall errors, which may have been minimized because most study physicians had very few LMC patients to recall and thus were more likely to recall accurately. Although we based our weighting procedures on insights that we gained from our past epidemiology and hospital study experience, our weighting system, like any weighting system, also could be a potential source of bias, and future research might indicate that the specific values used to adjust for co-management could be higher or lower.

## Conclusions

An accurate understanding of LMC’s treatment prevalence is vital to better understand the medical needs of this disease. Our study found that the treated annual prevalence of LMC in the United States was 79,920 unique patients across a host of relevant specialties, despite lack of FDA approved medical treatment. The study also found that a substantial proportion of LMC patients may require treatment throughout their lives. While rare, LMC still affects a substantial number of people in the United States who are being managed by one or more specialists. By better understanding the prevalence of people living with LMC in the United States who require treatment, efforts to both increase disease awareness and to identify underserved populations in need of potential new treatments can be better focused.

## Methods

We conducted a retrospective observational survey of a nationally representative sample of patient-care physicians in the United States most likely to treat LMC. Potential study participants were pediatricians, pediatric dermatologists, pediatric hematologists/oncologists, hematologists/oncologists, and dermatologists. Of the 420 physicians who visited the study website, 312 were randomly selected from national physician-specialty panels, and 108 were randomly selected from the corresponding specialties in the AMA Physician Masterfile. Selected physicians were sent invitations electronically to participate in a brief national study of an unnamed rare patient condition that would be disclosed at the study website. The invitation also indicated that physicians would receive an honorarium regardless of whether they had any patients with the study condition. We used this procedure to avoid having physicians with no LMC patients from self-selecting out of the study without going to the study site, which in turn could bias study results.

Because the number of patient-care physicians in the US differs by specialty and subspecialty, we weighted study physician self-estimates of the number of LMC patients treated in the past 12 months to reflect the corresponding proportion in the national universe. Extreme outliers, data points that differ significantly from others in the corresponding variable, can cause serious errors in study estimations. Outliers were defined as cases that fell more than 1.5 box lengths (interquartile range lengths) from the lower or upper hinge of the box. To avoid such errors, we assigned the median value for the variable to the outlier [13].

In addition, evidence that we derived from our study and the literature indicates that LMC is a complex and heavily co-managed condition that usually requires a multidisciplinary physician team to treat. Thus, to obtain nationally representative LMC patient estimates, we weighted physician self-estimates of LMC patients treated to adjust and correct for the high degree of physician co-management. In cases where a patient’s LMC was co-managed by one or more different physicians, that patient would count as less than one (or a whole) LMC patient for each specialty/sub-specialty of the patient’s LMC co-managers in accordance with the weighting procedures below. The corresponding resulting fractions of LMC patients were added to the whole numbers for patients who were not co-comanaged to derive the metric LMCs/MD. This was multiplied by the total number of patient-care physicians in each of the corresponding co-managed specialties to derive the estimated total number of LMC patients (Table 2). We accomplished this co-management adjustment task by implementing the following weighting procedures:

- The proportion of self-estimated patients whose LMC was not co-managed by additional physicians received a weight of “1”.
- The proportion of self-estimated patients whose LMC was co-managed during the past 12 months was weighted as follows:
  - If a patient was co-managed by a study physician at a vascular center, hospital, or by a physician who was part of a specialized vascular team but not located at the center, we assumed such a patient also saw 3 other co-managing physicians during the year and therefore received a weight of 0.25.
  - If a patient was co-managed by a physician in any other circumstance, we assumed the patient saw 2 other co-managing physicians and therefore assigned a weight of 0.33.

A study physician’s probability of treating patients for LMC also may be affected by the physician’s total patient workload. Thus, we compared the self-estimated annual number of patients treated for LMC by the study physicians with the corresponding workloads of patient-care physicians nationally in the same specialty. No statistical difference was found between the study sample of physicians and the corresponding national universe when specialties are combined. However, such was not the case for a subset of study physicians. The self-estimated number of total unique patients seen during the past 12 months by study dermatologists (estimated 1556) was significantly higher than would be expected from the results of a national survey that measured annual household member usage of dermatologists (estimated 1247 per dermatologist) [14]. To correct for this possible overrepresentation of patient workload by dermatologists in the study, we assigned each study dermatologist’s estimate of the number of LMC patients treated a weight of 0.8014. For the other specialties in this study, specialty-specific national average patient volumes were not available, so it was not possible to compare or adjust results from our sample. However, this physician-based methodology and its variants have been successfully used to estimate the size of various patient cohorts on national and multi-national levels [15–17].

## Data Availability

The datasets generated and/or analyzed during the current study are available from the corresponding author on reasonable request.

## Abbreviations

LM: Lymphatic malformations
LMC: Lymphatic malformations with a cutaneous component
FDA: US Food and Drug Administration
US: United States
